# Influence of feeding systems and seasons on the basic composition and content of fat-soluble antioxidants and on the antioxidant activity of cow's milk

**DOI:** 10.5194/aab-67-421-2024

**Published:** 2024-08-29

**Authors:** Jolanta Król, Aneta Brodziak, Agnieszka Wawryniuk, Barbara Topyła

**Affiliations:** Department of Quality Assessment and Processing of Animal Products, Faculty of Animal Sciences and Bioeconomy, University of Life Sciences in Lublin, Akademicka 13, 20-950 Lublin, Poland

## Abstract

The aim of this study was to assess the quality of raw milk, with a special focus on the content of fat-soluble antioxidants and antioxidant activity, depending on the production system (intensive, traditional), production season (spring/summer, autumn/winter), and breed of cow (Polish Holstein–Friesian, Simmental). The basic chemical composition of milk, i.e., fat content, lactose, protein (including casein), and dry matter, as well as the somatic cell count (SCC), concentrations of fat-soluble vitamins (A, D ${}_{3}$ , and E), and total antioxidant status (TAS) were determined. It was shown that the breed of cow, production system, and season significantly influenced the levels of the analysed vitamins in the milk, thus determining its antioxidant status. A significantly richer source of lipophilic vitamins, regardless of the breed of cow, was milk obtained in the spring/summer season (season 2), with statistically significant differences ( p≤0.01 ) found only in the milk of cows from the traditional production system (system I) in which feeding in the spring/summer season was based on the pasture. The higher content of antioxidant vitamins resulted in an increase in the antioxidant potential of the milk. The use of pasture in milk production is, therefore, the optimal way to adapt the composition of milk to the needs of modern consumers while ensuring proper animal welfare. This is also supported by consumers' growing interest in pasture-derived dairy products, as outdoor pasture-based feeding is a natural system for animals.

## 1 Introduction

Recent years have seen an increase in consumer interest in the health benefits of food. Antioxidants in particular are attracting attention due to their key role in maintaining the balance between pro-oxidative and antioxidant processes in the human body. Antioxidants significantly reduce or even inhibit oxidation processes taking place in living organisms. By scavenging and neutralizing reactive oxygen species, they support and enhance the body's defence mechanisms, which is particularly important for the prevention of certain diseases of civilization (Stobiecka et al., 2022). Milk contains many biologically active substances, including those with antioxidant properties. Oxidative processes can reduce the nutritional quality of milk and cause an increase in unwanted flavours, which negatively affect its psychosensory attributes. The main antioxidants in milk include fat-soluble vitamins, especially E, A, and D ${}_{3}$ (Cichosz et al., 2017; Magan et al., 2021; Vanitcharoen et al., 2018). Vitamins are an important group of organic substances with high biological activity that are essential for the growth and functioning of the body. They are involved in numerous crucial life processes, supporting metabolism and improving the activity of enzymes and catalytic proteins. Numerous studies have shown that vitamins used in their natural form are twice as effective as diet supplements in synthetic form (Clemente et al., 2015). Due to their antioxidant activity, they are used as pharmacological agents in the prevention and treatment of cancers of the colon, bladder, prostate, ovaries, breasts, and lungs (Kuczyńska et al., 2013). Vitamin E is also rightfully called the “vitamin of youth”, as it inhibits the cell ageing process and provides effective protection against the oxidation and degradation of epidermal lipids. Vitamin A is known mainly for its role in vision. Moreover, a significant reduction in the risk of stomach cancer has been observed in people consuming vitamin A, owing to its ability to induce apoptosis of cancer cells (Talib et al., 2024). Vitamin D ${}_{3}$ is also part of the non-enzymatic antioxidant system of milk. Its antioxidant activity involves inhibition of lipid peroxidation (Berretta et al., 2022). The human body requires only small amounts of vitamins, but a lack of them has a significant impact on health. Milk and dairy products are important sources of vitamins, primarily fat-soluble vitamins. It should be stressed that the composition of milk, including the content of vitamins, is determined by multiple factors both genetic and dietary. Numerous studies (Brodziak et al., 2012, 2018; Król et al., 2017, 2020; Kuczyńska et al., 2012) indicate that the breed of cow significantly influences the level of bioactive substances in the lipid fraction of milk. Another important factor determining the chemical composition of milk, including the content of bioactive substances, is diet (Clarke et al., 2020; Król et al., 2019; Puppel et al., 2023; Santa et al., 2022; Stobiecka et al., 2023), which is largely associated with the milk production system used on the farm and with the season of production. This is one of the possible ways to adjust the composition of milk to changing market demands, mainly the expectations of consumers and the dairy industry. For the consumer, the production system is a crucial factor influencing milk quality. It should be stressed that milk from grazing cows is more valued by consumers, not only because of its higher health-promoting quality, but also because the pasture system ensures adequate animal welfare (Kasapidou et al., 2023). Grazing cows can move freely and take plants directly from the pasture, including plants containing numerous active substances with a beneficial impact on physiological processes and thus on their health (Santa et al., 2022; Wróbel et al., 2023).

The aim of the study was to assess the quality of raw milk, with special focus on the content of fat-soluble antioxidants and antioxidant activity, depending on the following factors:

- the breed of cow (Polish Holstein–Friesian, Simmental);
- the production system (intensive, traditional); and
- the production season (spring/summer, autumn/winter).

## 2 Materials and methods

### 2.1 Research material

The study was carried out on three farms keeping cows of the Polish Holstein–Friesian and Simmental breeds. One of the farms used an intensive milk production system, while the other two used a traditional system. The characteristics of the farms are presented in Table 1. All of the farms were subject to use value assessment of dairy cattle and met the requirements for milk production defined in Commission Regulation (WE) No. 1662/2006 of 6 November 2006 amending Regulation (WE) No. 853/2004 of the European Parliament and of the Council laying down specific hygiene rules for food of animal origin. Raw milk samples were taken individually from each cow. In total, 288 milk samples were analysed – 150 from Polish Holstein–Friesian cows and 138 from Simmental cows.

**Table 1:** Characteristics of the farming systems included in the research.

Specification	Intensive farming (system PMR – partially mixed ration)	Traditional farming
Number of farms	1	2
Number of cows	100	30
Feeding of cows	Spring/summer season: maize silage + haylage – 29 kg grain meal – 4–5 kg Autumn/winter season: maize silage + haylage – 25 kg grain meal – 2–3 kg	Spring/summer season: pasture – ad libitum hay – 3 kg grain meal – 2 kg Autumn/winter season: haylage – 20 kg hay – 3 kg grain meal – 2–3 kg
Milking system	Milking parlour	Bucket milking system
Animal housing	Free-stall barns	Tie-stall barns

Milk samples were collected once a month. The period from September to March was designated autumn/winter (season 1), and the period from May to August was designated spring/summer (season 2).

Data on the milk yield of cows were obtained from documentation kept by the Polish Federation of Cattle Breeders and Dairy Farmers.

### 2.2 Methods

Milk samples from individual cows were collected into sterile 250 mL containers. The samples were transported to the laboratory under refrigerated conditions. Each raw milk sample was analysed for the following: chemical composition, i.e. content of fat, protein, lactose, and dry matter, with the Infrared Milk Analyzer (Bentley, USA); casein content, according to AOAC (2000); and somatic cell count (SCC) by flow cytometry using the Somacount 150 (Bentley, USA).

The reversed-phase high-performance liquid chromatography (RP–HPLC) method was used to determine the concentrations of the fat-soluble vitamins A, D ${}_{3}$ , and E. All samples were prepared using the Röse–Gottlieb fat extraction method described by Hewavitharana et al. (1996). The separations were performed using a Pursuit XRs 3–C18 column with 150 mm length and 4.6 mm diameter (Varian, USA). The phase was a mixture of acetonitrile, methanol, water, and dichloromethane (Sigma, Germany), and the flow rate was set to 1 $\mathrm{mL}\;\min^{-1}$ . The standards were analysed under identical conditions. The following standard vitamin solutions were used: ( ± ) α -tocopherol (vitamin E) with ≥97 % purity (HPLC) – Lot MKCB1992V, cholecalciferol (vitamin D ${}_{3}$ ) with ≥98 % purity (HPLC) – Lot SLBx3929, and retinol (vitamin A) with ≥99 % purity (HPLC) – Lot 142270 (Sigma-Aldrich, Germany). Qualitative identification of each substance was based on analysis of the retention times read from individual chromatograms using Star 6.2 Chromatography Workstation software (Varian, USA). Quantitative analysis was carried out using the external standard method separately for each standard.

The total antioxidant status (TAS) was determined using Randox tests (Tecan Austria GmbH, Grödig, Austria). The analysis consisted of spectrophotometric measurements of the degree of colour change of the resulting reactive radical ABTS^®^ (2,2'–azino-di-(3-ethylbenzothiazoline sulfonate)) using a UV–Vis spectrophotometer at a wavelength of 600 nm. ABTS^®^ was incubated with peroxidase (metmyoglobin) and $H_{2}O_{2}$ to obtain the ABTS^®^ + a radical cation. It has a relatively stable blue–green colour, which was measured at the wavelength. The antioxidants present in the added sample reduced the intensity of the blue–green colour in proportion to their concentration.

$$\begin{array}{c} \mathrm{HX}-{\mathrm{Fe}}^{\mathrm{III}}+H_{2}O_{2}\to X-({\mathrm{Fe}}^{\mathrm{IV}}\;=\;0)+H_{2}O\\ {\text{ABTS}}^{\text{®}}+X-(\mathrm{FeIV}\;=\;0)\to {\text{ABTS}}^{{\text{®}}^{*}}++\mathrm{HX}-{\mathrm{Fe}}^{\mathrm{III}} \end{array}$$

$\mathrm{HX}-{\mathrm{Fe}}^{\mathrm{III}}$ is metmyoglobin, $X-({\mathrm{Fe}}^{\mathrm{IV}}\;=\;0)$ + $H_{2}O$ is ferrimyoglobin, and ABTS^®^ is 2,2'-azino-di-(3-ethylbenzothiazoline sulfonate).

### 2.3 Statistical analysis

Statistical analysis of the results was performed using Statistica ver. 13.1 (2016) using one-way and multi-variate analyses of variance. Significant differences between means in groups were determined using the Tukey test for unequal sample sizes at significance levels of p≤0.05 and p≤0.01 .

## 3 Results and discussion

### 3.1 Daily yield and basic chemical composition of milk

A significant factor determining the profitability of farms keeping cows is their productivity. The data in Table 2 indicate that cows kept on farms focusing on intensive milk production had a significantly higher daily yield (of about 35 %) than cows from low-input farms using a traditional feeding system. The difference in favour of the intensive system amounted to 9.26 kg of milk in the case of the Holstein–Friesian breed and 7.1 kg for the Simmental breed. These results are confirmed in studies by other authors (Brodziak et al., 2012; Król et al., 2017; Litwińczuk et al., 2015). Irrespective of the breed of cow and the production system, cows produced more milk in the spring/summer season than in the autumn/winter season. One factor differentiating milk yield in cows is seasonal differences in feed quality. According to Auldist et al. (2013), the main fodder which increases milk yield in summer is pasture forage. In the present study, greater differences in milk production between the seasons (in favour of the pasture season) were noted in the traditional system, in which feeding in the spring/summer season was based mainly on pasture. In comparison to autumn/winter, in spring/summer the yield of cows kept in traditional systems was about 16.5 % higher for the Holstein–Friesian breed and more than 28 % higher in the case of the Simmental breed. No significant differences were noted in the intensive system, in which the diet did not change according to the season. The results of the present study pertaining to the effect of the season on the productivity of cows are in agreement with the findings of Brodziak et al. (2012). According to the authors, the daily yield of both Holstein–Friesian and Simmental cows was statistically significantly ( p≤0.01 ) higher in the spring/summer season. Król et al. (2016) compared the productivity of Simmental cows in different housing systems and seasons and reported a pronounced difference in the amount of milk obtained in favour of herds kept in an intensive system in the spring/summer season. In the autumn/winter season, the daily yield of these cows was lower by 5.7 kg of milk. Milk obtained in the spring/summer season, irrespective of the production system, had a significantly lower content of fat and protein, including casein. The highest content of these constituents was noted in the milk obtained in the intensive system, and the differences between the seasons were the smallest in this system. In the traditional system, seasonal differences, in favour of the spring/summer (pasture) season, were much greater and statistically significant ( p≤0.01 ). The milk of Holstein–Friesian cows obtained in the traditional system in the autumn/winter season had significantly higher contents of fat (by 0.24 p.p. – percentage points), protein (by 0.16 p.p.), casein (by 0.09 p.p.), and lactose (0.08 p.p.) in comparison to the spring/summer season. Significant seasonal differences were also shown for milk produced by Simmental cows. As in the case of the Holstein–Friesian breed, they were only obtained for the traditional system, due to the seasonal differences in feeding in this system. Milk obtained in the spring/summer season had higher contents of fat by 0.20 p.p., protein by 0.15 p.p., and casein by 0.13 p.p. than milk obtained in the autumn/winter season. This is confirmed in research by other authors (Cermanova et al., 2011; Morales-Almaráz et al., 2011), who showed a lower content of protein, including casein, in milk from pasture-fed cows. Brodziak et al. (2012) also reported a more favourable chemical composition of milk obtained in the autumn/winter season from both breeds analysed (Simmental and Holstein–Friesian). High seasonal variability in the content of basic milk constituents was reported by Heck et al. (2009), who assessed raw milk produced on Dutch farms. The protein content ranged from 3.21 g per 100 g of milk in June to 3.38 g per 100 g in December, while the fat content ranged from 4.10 to 4.57 g per 100 g in the same months. The authors attribute the lower protein content in milk in the pasture season to the lower proportion of concentrate feed in the diet in comparison to winter. Green forage is richer in fibre and poorer in starch, which leads to a reduction in the production of propionic acid (the main glucose precursor) in the rumen and thus to a reduction in protein content in milk. Auldist et al. (2013), on the other hand, showed that the protein content in milk increases during pasture feeding. In the present study, the milk of cows kept in the intensive system, in both the Holstein–Friesian and Simmental breeds, had a higher content of basic nutrients, including protein. It should be noted that the milk of Simmental cows had a significantly higher crude protein content, irrespective of the production system (3.39 % in the traditional system and 3.86 % in the intensive system), and a higher casein content (2.72 % and 2.98 %, respectively) in comparison to Holstein–Friesian cows (crude protein 3.26 % and 3.66 %, respectively; casein 2.56 % and 2.76 %, respectively). The reverse pattern was noted for fat. Milk from Simmental cows had lower crude fat contents by about 0.4 p.p. in the traditional system and 0.2 p.p. in the intensive system compared to milk from Holstein–Friesian cows. It should be added that statistically significant differences ( p≤0.01 ) were noted for the traditional system. In the intensive system, no significant seasonal differences were shown for the content of milk constituents for either the Holstein–Friesian breed or the Simmental breed. In a study by Sobotka et al. (2011), the use of a total mixed ration (TMR) feeding system resulted in a significantly higher fat and protein content in milk than in the case of traditional feeding, in both summer and winter. The TMR system was also shown to have a beneficial effect on the milk yield of cows and the hygienic quality of milk. The content of the nutrients remained stable in all the production systems, except for a somewhat higher level of fat in organic milk. In the study by Król et al. (2019), as the share of silage and industrial feed in the cows' ration increased, the percentage of basic milk components increased.

**Table 2:** Milk yield of cows and the basic chemical composition of milk from Simmental and Holstein–Friesian cows, taking into account the production system and season (mean, SD).

Parameter	System	Simmental			Holstein–Friesian			Factor influence		
		Season 1	Season 2	Total	Season 1	Season 2	Total	Breed	Season	System
		n=70	n=68	n=138	n=76	n=74	n=150			
Milk yield (kg)	I	13.05	16.79	14.70	15.34	17.07	16.07	*	*	**
		4.30	4.22	4.28	5.71	5.94	5.86			
	II	21.18	22.52	21.80	24.91	25.85	25.33	*	–	
		4.95	3.81	5.82	8.26	7.83	8.02			
Dry matter (%)	I	12.66	12.42	12.54	13.36	12.85	13.15	**	**	*
		0.73	0.56	0.66	0.91	0.98	0.97			
	II	13.53	13.40	13.46	14.11	13.00	13.61	*	–	
		0.94	0.64	0.80	1.07	0.85	1.12			
Total protein (%)	I	3.45	3.30	3.39	3.39	3.23	3.26	*	*	**
		0.41	0.46	0.28	0.29	0.27	0.44			
	II	3.90	3.82	3.86	3.71	3.62	3.66	**	–	
		0.35	0.29	0.32	0.46	0.41	0.50			
Casein (%)	I	2.78	2.65	2.72	2.66	2.57	2.56	**	*	*
		0.34	0.38	0.29	0.23	0.34	0.36			
	II	2.92	3.04	2.98	2.80	2.73	2.76	**	–	
		0.24	0.25	0.24	0.31	0.38	0.36			
Fat (%)	I	4.02	3.82	3.93	4.45	4.21	4.35	**	**	*
		0.46	0.36	0.42	0.61	0.60	0.62			
	II	4.35	4.33	4.34	4.61	4.51	4.54	*	–	
		0.32	0.41	0.36	0.56	0.50	0.63			
Lactose (%)	I	4.71	4.72	4.71	4.81	4.73	4.76	–	*	–
		0.20	0.29	0.24	0.28	0.26	0.28			
	II	4.63	4.59	4.61	4.74	4.79	4.75	–	–	
		0.43	0.26	0.35	0.37	0.19	0.30			

n – number of milk samples; System I – traditional system; System II – intensive system; Season 1 – autumn/winter season; Season 2 – spring/summer season; **, * – statistically significant differences: * – at p≤0.05 , ** – at p≤0.01 .

### 3.2 Fat-soluble antioxidants

Analysis of the effect of the factors analysed on the content of fat-soluble vitamins in milk showed that the milk of cows kept in a traditional system, in both the Holstein–Friesian and Simmental breeds, had higher contents of fat-soluble vitamins than the milk of cows of the same breeds kept in the intensive system. The content of fat-soluble vitamins in the milk of cows kept in a traditional system was higher on average by 33 % for vitamin A, 25 % for vitamin D ${}_{3}$ , and 43 % for vitamin E. It is worth stressing that the milk of cows kept in a traditional system at the same time had lower concentrations of basic nutrients, including fat. Irrespective of the production system, the content of vitamins was higher in the spring/summer season, although the differences were statistically significant ( p≤0.01 ) only in the case of the traditional production system. The milk of both Holstein–Friesian and Simmental cows obtained in the spring/summer season was a richer source of fat-soluble vitamins. Milk produced by Holstein–Friesian cows in the spring/summer season had about 50 % higher content of vitamin E and 60 % higher content of vitamins A and D ${}_{3}$ than milk from the autumn/winter season. The milk of Simmental cows in the pasture season contained 44 % more vitamin A, 62 % more vitamin D ${}_{3}$ , and as much as 72 % more vitamin E than milk obtained in the autumn/winter season. These differences were due to the different diet of cows in this system, which was mainly pasture-based in the spring/summer season. Numerous studies indicate higher concentrations of vitamins A, D ${}_{3}$ , and E in the milk of grazing cows (Butler et al., 2008; Radkowska, 2013; Strusińska et al., 2010; Toledo and Andrén, 2003). Fresh pasture sward has significantly higher contents of vitamins E and A than preserved fodder (Nozière et al., 2006; Radkowska, 2013). A high proportion of silage in the diet of cows can decrease the concentrations of fat-soluble vitamins compared to green forage, because preservation of feedstuffs, especially by drying, can reduce the content of β -carotene. According to Butler et al. (2008), the higher levels of maize silage used on conventional farms are largely responsible for the lower content of antioxidant vitamins in milk. In addition, exposure of cows to the Sun while in the pasture strongly promotes synthesis of vitamin D (Gabryszuk et al., 2013). As a consequence, the milk of cows kept indoors year-round will have lower biological value than that of cows with access to pasture. Strusińska et al. (2010) analysed milk from grazing cows and cows fed a TMR diet containing maize silage and reported that the content of vitamin E was quadrupled, that of β -carotene was doubled, and that of vitamin A was 25 % higher in the milk of cows that grazed in the pasture. Santa et al. (2022) showed that the introduction of pasture (8 $h\;d^{-1}$ ) in combination with TMR had a positive effect on the concentrations of fat-soluble vitamins in milk. In comparison with milk from cows fed TMR alone, it had a significantly higher ( p<0.05 ) content of fat-soluble vitamins, i.e. more than twice the content of vitamins E and A (0.74 vs. 0.27 mg per 100 g ; 125.62 vs. 57.51 µg per 100 g) and about 60 % more β -carotene (0.69 vs. 0.41 µg per 100 g). Similar conclusions were drawn by other authors (Alves et al., 2011; Magan et al., 2021): the higher levels of maize silage used on conventional farms are largely responsible for the lower content of antioxidant vitamins in milk. In addition, exposure of cows to the Sun while in the pasture strongly promotes synthesis of vitamin D (Gabryszuk et al., 2013). The authors showed that milk from the summer feeding season, when the cows grazed in the pasture and additionally received maize silage and concentrate feed on one of the farms, was distinguished by a higher content of substances with antioxidant properties, mainly β -carotene and vitamin D ${}_{3}$ , of 78 % and 14 %, respectively, compared to milk from winter feeding. A study in the Netherlands also showed that milk obtained in the summer and early autumn (July–October) had a significantly higher content of retinol (8.8 $µg\;g^{-1}$ fat) and β -carotene (4.1 $µg\;g^{-1}$ fat); this was 25 % more than in milk obtained in winter and early spring (January–April) (Hulshof et al., 2006). Another important factor is the quality of the pasture and its proportion of beneficial plant species (Kuczyńska et al., 2013; Kuhnen et al., 2021; Nozière et al., 2006). Good pasture sward should consist of 30 % tall grasses, 50 % short grasses, 10 %–20 % legumes, and about 10 % herbs (Radkowska et al., 2018).

Brodziak et al. (2018) also observed relationships between the content of fat-soluble vitamins in milk and the cow maintenance system. The authors compared the bioactive status of the milk of Simmental cows from organic, traditional, and intensive systems and reported a significantly higher content of vitamins (while the fat content was significantly lower) in milk from farms using organic and traditional systems. The concentration of vitamin A was 35 % higher, the concentration of vitamin D ${}_{3}$ was 23 % higher, and the content of vitamin E was as much as 90 % higher than in milk from cows kept in the intensive system. In another study, Radkowska (2013) compared the content of vitamins A and E in the milk of Holstein–Friesian cows kept in different maintenance systems (free stall, with a cattle run, and pasture). The author showed that the content of both vitamins in the milk of cows kept exclusively in the cowshed and cows with access to cattle runs was similar: 0.329 and 0.334 $µg\;{\mathrm{mL}}^{-1}$ , respectively, for vitamin A and 0.970 and 0.974 $µg\;{\mathrm{mL}}^{-1}$ , respectively, for vitamin E. However, the group of cows using the pasture had significantly higher contents of vitamins A (0.458 $µg\;{\mathrm{mL}}^{-1}$ ) and E (1.185 $µg\;{\mathrm{mL}}^{-1}$ ).

In the present study, irrespective of the production system, the breed of cow significantly influenced the content of vitamins A ( p≤0.05 ) and E ( p≤0.01 ). Milk from Simmental cows had higher contents of these vitamins: in the case of vitamin A from 10 % in the intensive system to 15 % in the traditional system, and in the case of vitamin E from 20 % to 30 %, respectively (Table 3). However, there was no effect on the content of vitamin D ${}_{3}$ . The results of the present study are supported by Król et al. (2017). The authors compared four breeds of cows kept in an intensive system in Poland (Simmental, Holstein–Friesian, Montbéliarde, and Jersey) and showed that milk from Simmental cows contained on average 20 % more vitamin A and 10 % more vitamin E than milk from the other breeds, with the milk of Holstein–Friesian cows shown to be the poorest source of these vitamins. In another study by the authors (Król et al., 2020), milk from Simmental cows used for quark production was a richer source of vitamins A and E than milk from Holstein–Friesian cows. Ramalho et al. (2012) also showed that the breed of cow has a pronounced influence on the concentrations of fat-soluble vitamins in milk, which according to the authors should be used in genetic selection. They found that milk from the indigenous Minhota breed had a significantly higher content of vitamin A (9.98 $µg\;g^{-1}$ fat, about 20 % higher), α -tocopherol (32.79 $µg\;g^{-1}$ fat, twice as high), and β -carotene (3.60 $µg\;g^{-1}$ fat, more than twice as high) than milk from Holstein cows. No significant differences were shown only in the case of the content of vitamin D ${}_{3}$ (Minhota 0.11 $µg\;g^{-1}$ fat and Holstein 0.10 $µg\;g^{-1}$ fat). The authors showed no seasonal changes in the content of fat-soluble vitamins in the milk of Holstein–Friesian cows and only minor changes in the case of the Minhota breed (for monthly milk collection throughout the year). A study by Mogensen et al. (2012), conducted on organic farms with Holstein–Friesian and Danish Red cows, showed a significantly higher content of vitamin E in the milk of Holstein–Friesian cows (0.90 vs. 0.51 $µg\;{\mathrm{mL}}^{-1}$ ).

**Table 3:** Content of lipophilic vitamins in the milk of Simmental and Holstein–Friesian cows, taking into account the production system and season (mean, SD).

Parameter	System	Simmental			Holstein–Friesian			Factor influence		
		Season 1	Season 2	Total	Season 1	Season 2	Total	Breed	Season	System
		n=70	n=68	n=138	n=76	n=74	n=150			
Vitamin A ( $\mathrm{mg}\;L^{-1}$ )	I	0.371	0.520	0.449	0.308	0.534	0.391	*	**	**
		0.133	0.126	0.134	0.121	0.078	0.162			
	II	0.324	0.325	0.325	0.302	0.303	0.302	*	–	
		0.102	0.049	0.106	0.049	0.057	0.103			
Vitamin D ${}_{3}$ ( $µg\;L^{-1}$ )	I	0.496	0.808	0.646	0.478	0.770	0.611	–	**	*
		0.150	0.300	0.281	0.108	0.216	0.217			
	II	0.492	0.536	0.504	0.453	0.516	0.491	–	–	
		0.078	0.107	0.095	0.116	0.158	0.139			
Vitamin E ( $\mathrm{mg}\;L^{-1}$ )	I	1.418	2.507	1.941	1.266	1.863	1.519	**	**	**
		0.265	0.597	0.710	0.262	0.291	0.403			
	II	1.293	1.322	1.308	1.091	1.222	1.113	**	–	
		0.143	0.180	0.162	0.392	0.180	0.323			

n – number of milk samples; System I – traditional system; System II – intensive system; Season 1 – autumn/winter season; Season 2 – spring/summer season; **, * – statistically significant differences: * – at p≤0.05 , ** – at p≤0.01 .

### 3.3 Antioxidant activity

**Figure 1:**
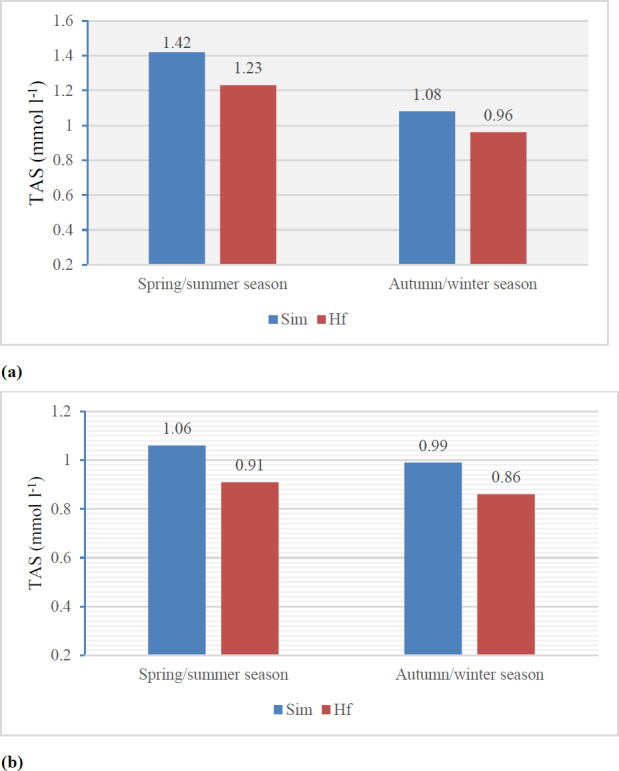
The total antioxidant status (TAS) of milk: **(a)** traditional system; **(b)** intensive system.

Research has shown that the use of pasture for feeding cows significantly influences the content of fat-soluble antioxidants in milk and thus its antioxidant activity (Santa et al., 2022; Stobiecka et al., 2022; Torre-Santos et al., 2020). According to Yilmaz-Ersan et al. (2018), differences in the total antioxidant potential of milk are explained by differences in its chemical composition, primarily the content of antioxidant compounds, including fat-soluble vitamins and whey proteins, which are a rich source of sulfur-containing amino acids (cysteine and methionine). In the present study as well, a higher content of antioxidants in milk translated to an increase in its antioxidant potential. Irrespective of the production system and season, the antioxidant activity of milk from Holstein–Friesian cows was significantly ( p≤0.05 ) lower than in the case of Simmental cows (Fig. 1a and b). The highest antioxidant activity was noted for milk obtained from Simmental cows during the pasture season (Fig. 1a). In a study by Kasapidou et al. (2023), the production season also had a significant effect ( p<0.05 ) on the antioxidant activity of milk, expressed as free-radical scavenging activity (DPPH – 2,2-diphenyl-1-picrylhydrazyl) and ferric-reducing antioxidant power (FRAP), with higher levels noted in spring and summer.

Kuhnen et al. (2014) found positive correlations between total phenol content (TPC) and FRAP values for milk samples (0.198, P<0.05 ) and milk FRAP and forage TPC values (0.344, P<0.05 ). According to the authors, the content of phenols in pasture vegetation can be used as a marker of milk production with greater antioxidant activity. In a subsequent study (Kuhnen et al., 2021), the authors pointed out that, through appropriate management in pasture-based milk production systems, the milk quality can be improved. Santa et al. (2022) reported that the content of fat-soluble antioxidants in milk was also associated with its antioxidant activity. In comparison with a TMR diet, milk from grazing Jersey cows had significantly higher contents of vitamins E (0.74 vs. 0.27 mg per 100 g) and A (125.62 vs. 57.51 µg per 100 g) and β -carotene (0.69 vs. 0.41 µg per 100 g), which translated to higher antioxidant activity in the milk (3.02 vs. 2.53 $µ\mathrm{mol}\;\mathrm{TE}\;{\mathrm{mL}}^{-1}$ ). Stobiecka et al. (2023) reported significant ( p≤0.01 ) positive correlation coefficients between the content of vitamins A and E and the total antioxidant potential of milk, which indicates that the content of these compounds largely determines the antioxidant potential of the milk. The highest correlation coefficients were noted between the total antioxidant potential and the content of vitamins A ( r=0.687 ) and E ( r=0.664 ). Studies by other authors (Kapusta et al., 2018; Puppel et al., 2017) also showed that milk which was richer in vitamins A and E had a greater antioxidant potential. It is more difficult to improve the antioxidant potential of milk in the case of intensive production using preserved feedstuffs (Król et al., 2019; Magan et al., 2021). In this case, studies (Mohammadabadi et al., 2023; Stobiecka et al., 2023) indicate that the antioxidant potential of milk can be increased by introducing various natural additives to the diet, mainly herbs. Owing to their high content of biologically active substances, they exert a positive effect on the functioning of the cow's body, which translates to the quality of the milk (Gabryszuk et al., 2013; Paskudska et al., 2018). According to Puppel et al. (2023), the use of cold-pressed linseed cake in winter allows the antioxidant potential of milk to be increased, thus eliminating disproportions in milk quality compared to the summer season when animals use the pasture.

## 4 Conclusion

To sum up, the breed of cow, production system, and season significantly influenced the level of vitamins in milk, thus determining its bioactive status. Irrespective of the production system, milk from Simmental cows was a significantly richer source of vitamins A and E. This was especially true of cows grazing in pasture, which was associated with traditional systems. The higher content of antioxidant substances in the milk translated to an increase in its antioxidant potential, which plays an important role in supporting mechanisms enabling the neutralization and scavenging of free radicals. In view of the above, it is worth considering the possibility of separate purchasing and processing of milk with a higher antioxidant potential, due to its potentially beneficial impact on consumer health. In addition, products made from this milk could be more competitive on the market. For many consumers, information on the origin of milk from grazing cows will be an important factor when choosing a product.

## Data Availability

The code and datasets used and analysed during this study are available from the corresponding author upon reasonable request.
